# Resistance of Aluminide Coatings on Austenitic Stainless Steel in a Nitriding Atmosphere

**DOI:** 10.3390/ma15010162

**Published:** 2021-12-27

**Authors:** Karolina Wierzbowska, Agnieszka Elżbieta Kochmańska, Paweł Kochmański

**Affiliations:** Faculty of Mechanical Engineering and Mechatronics, West Pomeranian University of Technology in Szczecin, Av. Piastow 17, 70-310 Szczecin, Poland; agnieszka.kochmanska@zut.edu.pl (A.E.K.); pawel.kochmanski@zut.edu.pl (P.K.)

**Keywords:** slurry cementation, aluminide coatings, nitriding, stainless steel, high temperature corrosion

## Abstract

A new slurry cementation method was used to produce silicide-aluminide protective coatings on austenitic stainless steel 1.4541. The slurry cementation processes were carried out at temperatures of 800 and 1000 °C for 2 h with and without an additional oxidation process at a temperature of 1000 °C for 5 min. The microstructure and thickness of the coatings were studied by scanning electron microscopy (SEM). The intention was to produce coatings that would increase the heat resistance of the steel in a nitriding atmosphere. For this reason, the produced coatings were subjected to gas nitriding at a temperature of 550–570 °C in an atmosphere containing from 40 to 60% of ammonia. The nitriding was carried out using four time steps: 16, 51, 124, and 200 h, and microstructural observations using SEM were performed after each step. Analysis of the chemical composition of the aluminide coatings and reference sample was performed using wavelength (WDS) and energy (EDS) dispersive X-ray microanalysis, and phase analysis was carried out using X-ray diffraction (XRD). The resistance of the aluminide coatings in the nitriding atmosphere was found to depend strongly on the phase composition of the coating. The greatest increase in resistance to gas corrosion under nitriding atmosphere conditions was achieved using a manufacturing temperature of 1000 °C.

## 1. Introduction

Austenitic stainless steels are the most common alloys applied in refining, petrochemical plants, and thermo-chemical treatment furnace equipment for applications such as retorts, pressure vessels, piping components, manifolds, cyclones, fittings, and valves [1,2,3,4]. Austenitic stainless steels containing 18 wt.%. chromium and a minimum of 8 wt.% nickel are resistant to corrosion in air up to a maximum temperature of 850 °C [5,6]. However, the corrosion resistance in atmospheres containing aggressive substances such as ammonia or carbon monoxide is much lower due to the difficulties in rebuilding the passive layer of chromium oxide [7].

One of the most crucial problems in industries dealing with thermo-chemical treatment (e.g., carburizing or nitriding) is a limited lifetime of furnace equipment resulting from hot corrosion [8]. Nitridation is a problem that occurs when steels are exposed to an ammonia environment at elevated temperatures, and can also result from nitrogen atmospheres, especially under reducing conditions and high temperatures [9]. Nitridation occurs when chromium and iron (also other elements) from steels combine with nitrogen to form embrittling nitrides on the surface. An interesting approach to circumvent the above problems is the use of surface alloying. In this approach, a highly alloyed and highly resistant surface layer is produced, whereas the bulk substrate composition and properties remain unchanged [10].

To extend the life of various parts in aggressive atmospheres containing, for example, ammonia, protective coatings containing titanium [11] can be used. The application of adsorbed sulfur or a protective oxide (e.g., Cr_2_O_3_, or the much more thermodynamically stable Al_2_O_3_) to the alloy surface can also be used to protect against hot corrosion and consequently against metal dusting in an atmosphere containing ammonia [12]. However, if the protective layer is damaged by chipping or cracking, internal nitridation of the alloy may occur [8].

Increasing the corrosion resistance of steel can also be achieved by the use of methods such as pack-cementation [13,14], physical vapor deposition (PVD) [15,16], and multi-functional CVD-PVD [17]. These are the most common surface engineering methods that allow aluminum to be incorporated into steel. The inward diffusion of aluminum and the outward diffusion of iron, nickel, and chromium result in the formation of intermetallic phases with very interesting protective properties.

A new and novel means to increase the hot corrosion resistance is the use of an aluminide diffusion coating produced by slurry cementation [18]. In this way, intermetallic phases including FeAl, Fe_3_Al, NiAl, and Ni_3_Al [18,19] are formed.

Aluminides contain from 10 to 30 wt.% amount of aluminum, a significantly higher amount of Al than in common alloys or superalloys. It is known that nickel aluminides and iron aluminides are resistant to oxidation and carburization up to temperatures of around 1000 °C [20,21]. However, studies describing the resistance of aluminides to nitridation are not widely reported [22]. Recently, nitriding of iron aluminides to increase the hardness of such phases or alloys based on these phases has been reported with growing frequency [23,24,25,26].

This work investigates the resistance of the silicide-aluminide coatings produced by slurry cementation on austenitic stainless steel 1.4541 under gas nitriding conditions. The use of slurries to produce protective coatings has many advantages over other technologies such as pack cementation [27]. For example, a shorter thermal cycle of coating application due to rapid heating and cooling of the treated component; the possibility of local aluminizing; and the possibility of applying coatings to large parts with complicated shapes. It is also important to note that the slurry method is relatively low-cost due to the low consumption of the powder material. Several different elements can be co-deposited using this method, so not only can aluminum be incorporated, but also, for example, silicon. Usually, the slurry contains powders of metals and an organic binder [28]. In this study, an inorganic binder instead of an organic one was applied, obviating the need for additional curing by heating in order to remove the organic binder. The use of an inorganic binder enables annealing directly at the temperature at which the diffusion process takes place. Moreover, one of the most important physical properties of the binder used (i.e., soluble silicate solutions (water glass)) is high viscosity [19], which provides appropriate properties for slurry application by affecting the viscosity of the ready-to-use slurry. This ensures that the applied slurry achieves very good mechanical properties after drying (evaporation of water), especially hardness. This is a very important aspect in the technological process of this method because there is very little possibility of damaging the dried slurry on the workpiece. In addition, the slurry is very easily removed after annealing (coating formation).

These innovative silicide-aluminide coatings were produced and exposed to gas nitriding within a temperature range of 550–570 °C in an atmosphere containing 40–60% ammonia for 200 h, after which the corrosion resistance was assessed. Additionally, some coated samples were oxidized prior to nitridation in order to create a protective layer of aluminum oxide on their surface. On the basis of the results, a selection could be made of coatings with specific phase composition to give the best performance in terms of resistance to the nitriding atmosphere. The article also presents the process of high-temperature corrosion of a stainless steel in a nitriding atmosphere.

## 2. Materials and Methods

**Figure 1:**
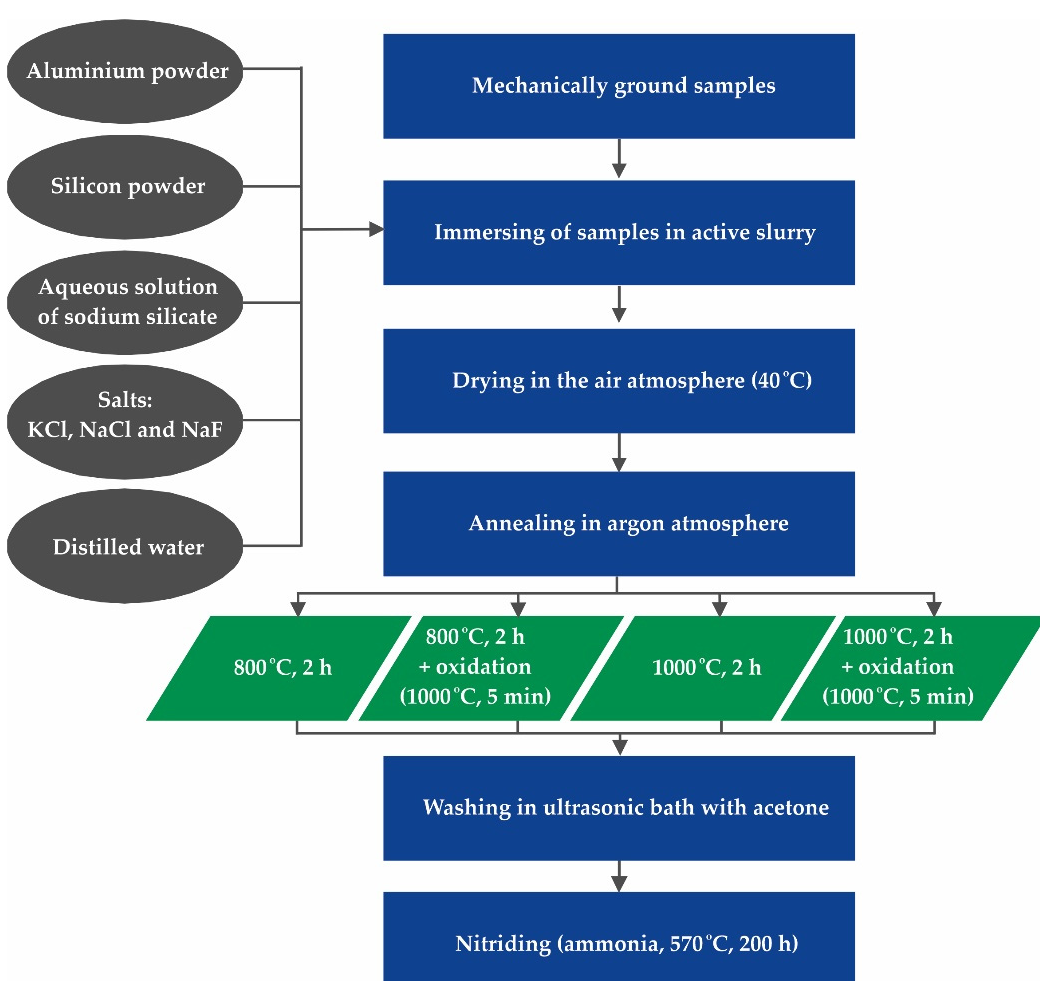
Scheme of the experimental protocol.

The diagram of the protocol of the experiment is shown in Figure 1.

### 2.1. Materials

The coatings were produced on austenitic stainless steel 1.4541 (X6CrNiTi18-10) with the chemical composition [wt.%]: C—0.1; Si—0.3; Cr—18.6; Ni—8.3, and Fe—balance. The samples with dimensions of 20 mm × 10 mm × 6 mm were mechanically ground (Ra = 0.1 µm) and cleaned in an acetone bath before immersing in the slurry. In this work, an active slurry was prepared containing aluminum powder type AG 160 (granularity up to 75 µm, minimum aluminum content 99.7%, Benda-Lutz, Skawina, Poland); silicon powder (granularity 200–250 µm, minimum silicon content 99%, Chemical Worldwide Business SA, Słupca, Poland); an aqueous solution of sodium silicate (with molar ratio 3 and the dynamic viscosity of 0.05 Pa∙s; Hartim Chemikals & Solutions) performing the role of a binder; a mixture of halogens (KCl, NaCl, NaF molten at a temperature of 600 °C, Hartim Chemikals & Solutions, Kraków, Poland); distilled water. These components were used in the weight ratio 180:20:30:19:100. All components were carefully manually mixed until the slurry formed a suspension. Samples of the steel were immersed in the slurry and dried in an air atmosphere (Figure 1—40 °C, minimum 30 min), after which the density of the slurry on the surface was 0.3 g/cm^2^. A weight measurement was used after each dipping to determine the exact amount of slurry applied. The samples, covered with the dried slurry, were annealed in an inert atmosphere of argon. Coatings were produced under the four combinations of conditions given in Table 1. The heating rate was 20 °C per minute and the cooling was carried out with the furnace.

After annealing, the slurry residues were easily mechanically removed from the surface, and the samples subsequently washed in acetone in an ultrasonic bath. All coated samples were gently ground and polished to remove only the surface roughness and expose the top layer of the coating, in order to facilitate and simplify the further characterization of changes occurring during high-temperature corrosion in a nitriding atmosphere. The roughness Ra of the surface was 0.1 µm.

After removing the residual slurry and grinding the surface, the samples with coatings No. 2 and 4 were oxidized at 1000 °C for 5 min. Coatings resistant to high-temperature corrosion most frequently rely on the formation of a dense, adherent alumina layer at the interface between the coating and the environment. Thus, the purpose of the additional oxidation was to form such an aluminum oxide top layer.

The samples were placed in an aggressive atmosphere of ammonia (gas nitriding furnace retort) at a temperature of 550–570 °C. The content of ammonia in the working atmosphere was maintained within the range 40–60%, the remainder being products of ammonia dissociation. The degree of ammonia dissociation was measured in the exhaust gas using a dissociation pipette. The conditions applied to test the resistance to nitridation corresponded to the practical operating conditions commonly used in thermo-chemical treatment. This process was stopped four times (after 16 h, 51 h, 124 h, and 200 h of nitriding) in order to monitor changes in the microstructure.

### 2.2. Methods

The surface of all samples was examined using a field emission scanning electron microscope (FE-SEM) Hitachi SU-70 (Hitachi, Naka, Japan) with an X-ray microanalysis UltraDry EDS detector (acceleration voltage 15 kV) and a Magnaray WDS detector, Thermo Scientific Noran System 7 (Madison, WI, USA). The WDS analysis was performed at an accelerating voltage of 10 kV and an electron beam current of approximately 20 nA using CrSc80, NiC80, and TAP diffracting crystals for nitrogen, carbon, and oxygen, respectively. The WDS quantitative analytical procedures were based on the following standards: Cr_2_N, WC, and Cr_2_O_3_ for nitrogen, carbon, and oxygen, respectively. The “PROZA” correction method was applied for WDS quantitative analysis and the estimated standard uncertainty for the WDS measurements was 0.1 at.% X-ray diffraction (XRD) phase analysis was performed using CuKα, using X-ray tube parameters of 35 kV and 45 mA and a Bragg–Brentano geometry (X’Pert–PRO, Panalytical, Almelo, The Netherlands). The applied step of the goniometer was 0.05, and the acquisition time was 200 s. The acquired data were processed using X’Pert HighScore (v. 2.2.1) software provided by Panalytical.

## 3. Results

### 3.1. Coatings as Produced

**Figure 2:**
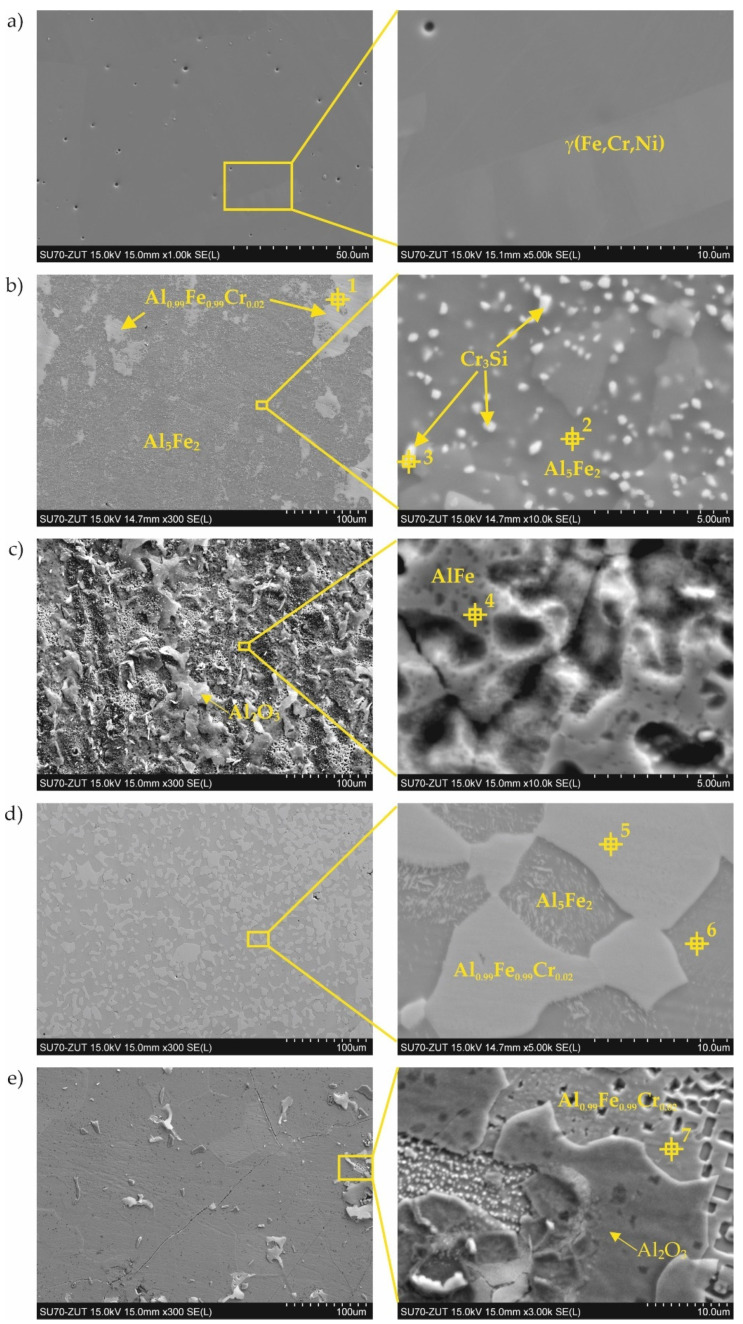
Surface of 1.4541 steel: (**a**) uncoated; (**b**) with coating No. 1 (800 °C, 2 h); (**c**) with coating No. 2 (800 °C, 2 h + oxidation 1000 °C); (**d**) with coating No. 3 (1000 °C, 2 h); (**e**) with coating No. 4 (1000 °C, 2 h + oxidation 1000 °C).

**Figure 3:**
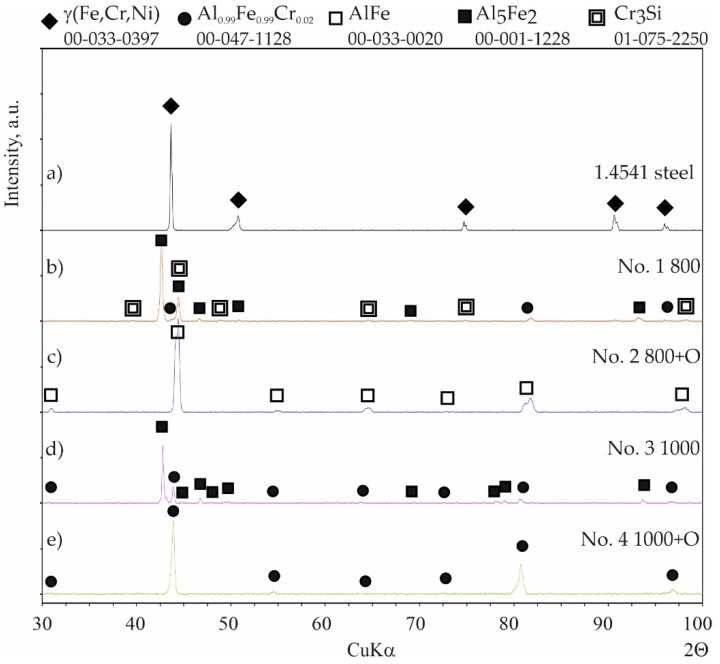
Diffraction patterns of 1.4541 steel: (**a**) uncoated; (**b**) with coating No. 1 (800 °C, 2 h); (**c**) with coating No. 2 (800 °C, 2 h + oxidation 1000 °C); (**d**) with coating No. 3 (1000 °C, 2 h); (**e**) with coating No. 4 (1000 °C, 2 h + oxidation 1000 °C).

Coatings were successfully produced for each set of assumed technological parameters. Figure 2 shows the results of the SEM studies of the surfaces of the uncoated steel and all types of coatings before nitriding, and the XRD phase analysis results are shown in Figure 3. The elemental chemical composition measured using EDS X-ray microanalysis is presented in Table 2.

The surface of the reference sample (i.e., 1.4541 steel) without the coating was smooth and uniform (Figure 2a).

In the case of coating No. 1 (800 °C, 2 h), two types of areas were observed in the microstructure (Figure 2b). The lighter of these was identified on the basis of XRD analysis as the intermetallic phase Al_0.99_Fe_0.99_Cr_0.02_ (Figure 3, Table 2—point 1). The darker areas formed mainly from the Al_5_Fe_2_ phase (Table 2—point 2), however, a peak from Cr_3_Si (Figure 3, Table 2—point 3) was also observed in the XRD diffraction pattern.

A different (more developed) surface morphology was observed on coating No. 2 (Figure 2c). XRD examination revealed the presence of AlFe intermetallic phase as the main phase component (Figure 3). However, X-ray microanalysis results indicated that aluminum oxides were also present on the surface (Table 2—point 4). This alumina layer was discontinuous (Figure 2c—left side).

A two-phase microstructure was observed on the surface of coating No. 3 (Figure 2d). The components, identified by XRD combined with SEM-EDS, were: Al_0.99_Fe_0.99_Cr_0.02_ intermetallic phase (Table 2—point 5) and Al_5_Fe_2_ phase (Table 2—point 6). The manufacturing temperature of 1000 °C caused the Cr_3_Si phase not to form, as was the case with coating No. 1. Chromium was present in the Al_0.99_Fe_0.99_Cr_0.02_ phase and this was dissolved in the Al_5_Fe_2_ phase, while silicon was dissolved at this temperature in both phases identified in the coating.

In the case of coating No. 4 (1000 °C, 2 h with oxidation), only one phase Al_0.99_Fe_0.99_Cr_0.02_ (Figure 2e, Table 2—point 7) was identified by XRD (Figure 3). Additional annealing at 1000 °C resulted in the formation of aluminum oxide on the surface. Numerous cavities were also observed on the surface (Figure 2e—right side).

**Figure 4:**
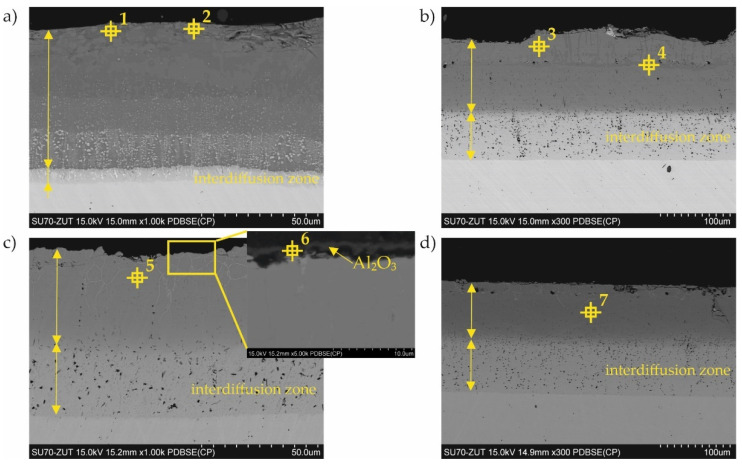
The microstructure (BEI) of the cross-section of Al–Si coatings obtained at (**a**) 800 °C for 2 h (No. 1); (**b**) 1000 °C for 2 h (No. 3); (**c**) 800 °C for 2 h and oxidated at 1000 °C (No. 2); (**d**) 1000 °C for 2 h and oxidated at 1000 °C (No. 4).

A typical example of the cross-sectional microstructure of the aluminide-silicide coatings obtained at different temperatures is shown in Figure 4. Generally, the coatings consisted of two zones: an outer zone containing intermetallic phases and an interdiffusion zone near the substrate. The average thickness of the aluminide-silicide coating produced at 800 and 1000 °C was 66 µm ± 11 and 180 µm ± 16, respectively, and the average thickness of coatings produced at 800 and 1000 °C and additionally oxidated was 77 µm ± 5 and 181 µm ± 2, respectively Significant differences were observed in the case of coatings on samples No. 1 and No. 3. Oxidation at 1000 °C caused the coating to increase by 10 µm, and in particular, the interdiffusion zone was from about 5 to 30 µm (Figure 4a,c).

The chemical composition is additionally shown in the cross-section of the coatings in Table 3. The points shown in this table are marked in Figure 4. The results of the chemical analysis acquired from the surface (Table 2) and performed on the cross-section (Table 3) of the coatings were consistent in terms of the special resolution of the X-ray microanalysis.

### 3.2. Nitriding Test

**Figure 5:**
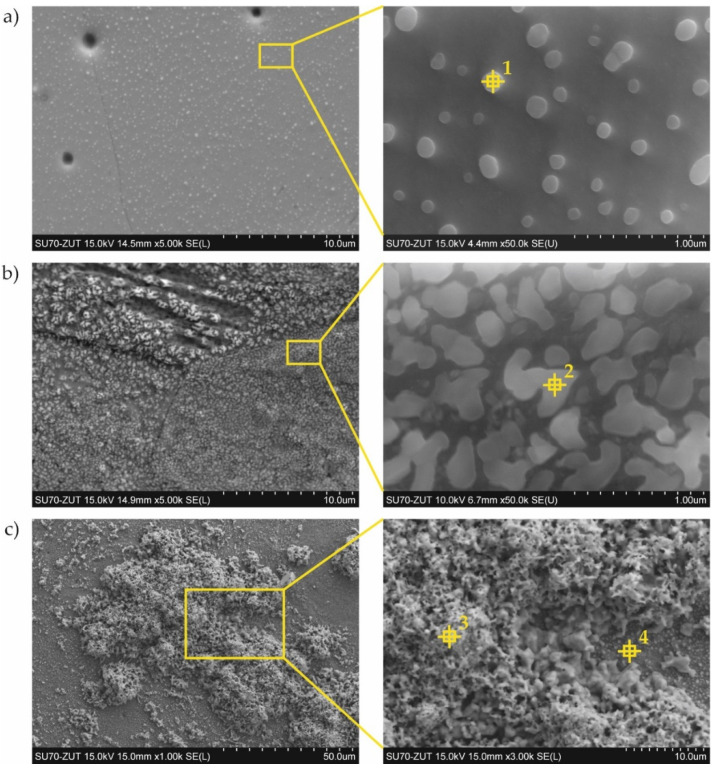
Surface of the uncoated, reference sample of 1.4541 steel after (**a**) 51 h, (**b**) 124 h, and (**c**) 200 h of nitriding.

**Figure 6:**
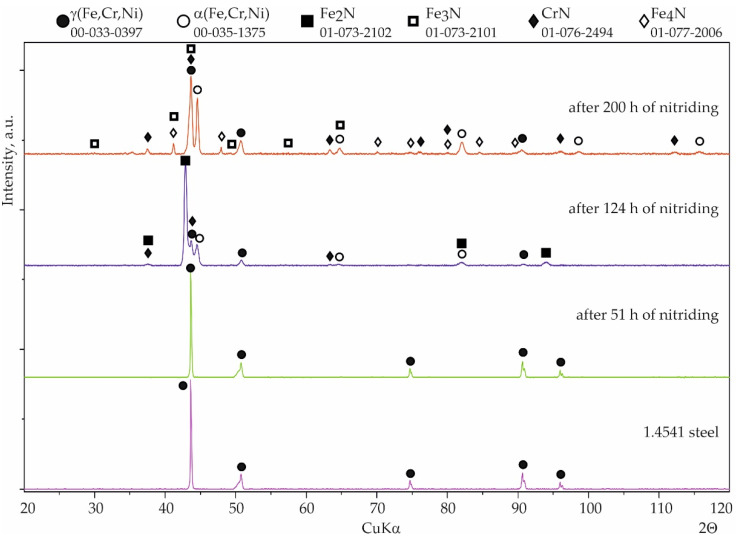
Qualitative X-ray structure analysis (XRD) of the 1.4541 steel; from the bottom: in the initial state; after 51 h of nitriding; after 124 h of nitriding; and after 200 h of nitriding.

Inspection of the surfaces of the reference samples without coatings after the nitridation resistance test showed no significant changes after 16 h of nitriding. After 51 h of nitriding, numerous nuclei of nitrides were observed on the surface of the sample (Figure 5a), indicating the formation of corrosion centers. Although the XRD results did not detect iron nitrides due to the very small amount and thickness of the precipitates, WDS point analysis indicated an increased amount of nitrogen within the limits 3–16 at.% (Table 4). This confirmed that austenitic stainless steel 1.4541 is not resistant to an aggressive atmosphere containing ammonia at elevated temperatures. After 124 h of exposure to the nitriding atmosphere, the iron nitrides had become much more developed on the surface. The corrosion appeared over the entire surface, and this was clearly visible in the SEM images (Figure 5b). The nitrogen content, determined by the WDS point analysis, was from 7 to 19.5 at.%. XRD phase analysis revealed the presence of nitrides of ε(Fe_2_N, Fe_3_N) and γ’(Fe_4_N) types and also CrN (Figure 6). Moreover, the austenite structure had been partially transformed into the more stable alloy ferrite α(Fe, Cr, Ni); this has also been reported elsewhere [29]. Significant growth of iron and chromium nitrides was observed after 200 h of nitriding (Figure 5c). Two types of surface morphologies could be distinguished: a relatively smooth layer composed mainly of CrN arranged linearly in longitudinal strands, and a second layer formed from γ’(Fe_4_N) with a highly developed spongy surface. The development of such a spongy morphology of the iron nitrides leads to a very strong increase in the catalytic properties for the dissociation of ammonia, which causes a local decrease in the nitriding potential. In comparison to the results from the 124 h nitriding time, the diffraction peaks from ε nitrides were no longer detected, most likely due to the transformation of this nitride into γ’(Fe_4_N), which may have been the result of the aforementioned decrease in the nitriding potential. The intensity of the diffraction peaks attributed to α(Fe, Cr, Ni) ferrite was increased, which could indicate further decomposition of the steel.

The resistance to the ammonia-containing atmosphere of the aluminide-silicide coatings was evaluated after the same time periods as that of the uncoated steel (i.e., 16 h, 51 h, 124 h, and 200 h of nitriding). SEM observations confirmed the absence of any corrosion nuclei after 16 h and 51 h of nitriding.

**Figure 7:**
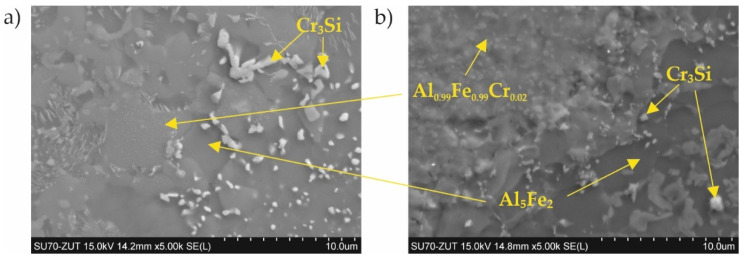
Microstructure of the sample with the coating manufactured at 800 °C, 2 h after (**a**) 51 h of nitriding; (**b**) 200 h of nitriding.

**Figure 8:**
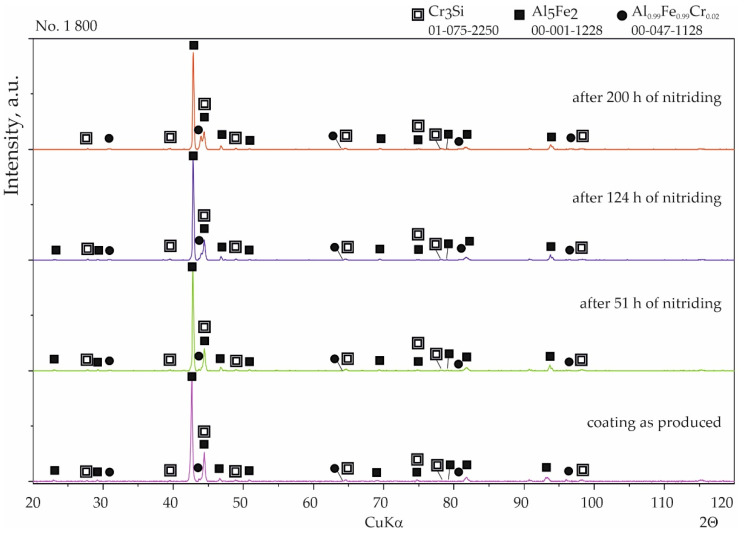
Qualitative X-ray structure analysis (XRD) of the Al–Si coating on 1.4541 steel produced at 800 °C and 2 h; from the bottom: in the initial state; after 51 h of nitriding; after 124 h of nitriding; and after 200 h of nitriding.

With coating No. 1 (800 °C, 2 h), precipitates of nitrides were not observed after 51 h and also after 124 h of nitriding. WDS analysis after 51 h showed a negligible amount of nitrogen (0.1 at.%), comparable to the measurement uncertainty, indicating the absence of any corrosion products. The nitrogen content was at a similar level, 0.2–0.1 at.%, after 200 h of nitriding (Figure 7b). Analysis of the phase composition by XRD of the surface of this coating showed no significant changes after all stages of nitriding (Figure 8).

**Figure 9:**
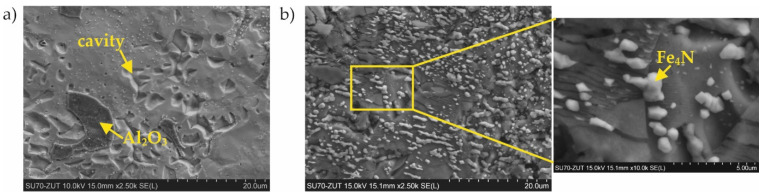
Microstructure of the sample with the coating manufactured in 800 °C, 2 h with additional oxidation in 1000 °C during 5 min after (**a**) 124 h and (**b**) 200 h of nitriding.

**Figure 10:**
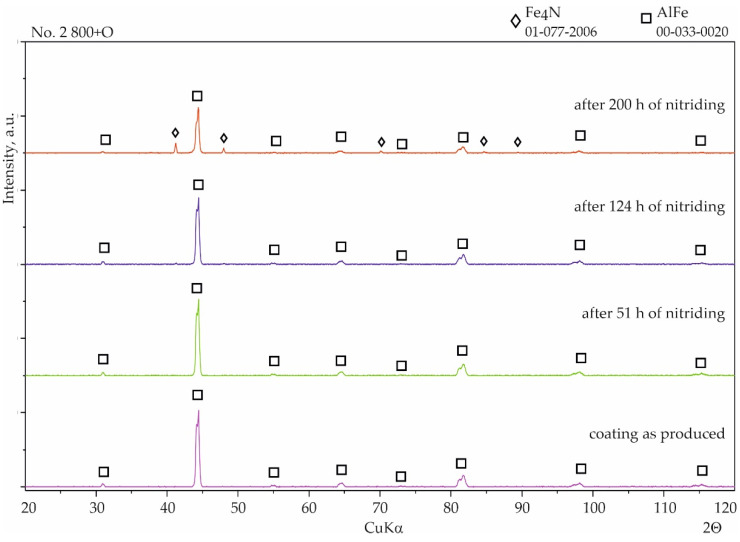
Qualitative X-ray structure analysis (XRD) of the Al–Si coating on 1.4541 steel produced at 800 °C with additional oxidation in 1000 °C during 5 min; from the bottom: in the initial state; after 51 h of nitriding; after 124 h of nitriding; and after 200 h of nitriding.

Considerable changes of structure were observed in the case of coating No. 2 (800 °C, 2 h with oxidation). After 124 h of nitriding, numerous cavities with regular shapes had appeared on the surface (Figure 9a). Minor precipitates were also visible on the surface and, as a result of the additional oxidation process, thin layers of aluminum oxide (Al_2_O_3_) were observed. However, this layer was discontinuous (Figure 9a). Despite the passive layer of aluminum oxide, numerous iron nitrides with a significantly developed area were observed on the surface. WDS analysis performed on areas of the surface without the Al_2_O_3_ layer indicated an average of 0.7 at.% nitrogen. After 200 h of nitriding, numerous precipitates of iron nitrides were observed, which had grown from between cracks or delaminated areas of the structure (Figure 9b) and WDS analysis showed that the nitrogen content had increased to approximately 8 at.% The microscopic SEM-WDS studies were confirmed by the results of the XRD phase analysis (Figure 10) (i.e., Fe_4_N nitrides were identified after 200 h of nitriding).

**Figure 11:**
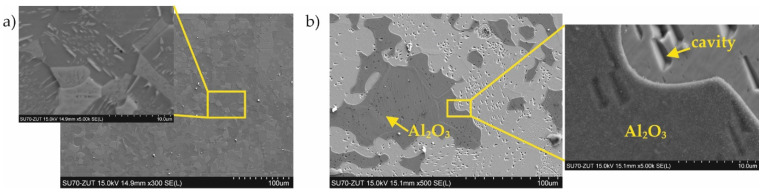
Microstructure of the sample with the coating manufactured in (**a**) 1000 °C, 2 h and (**b**) 1000 °C, 2 h with additional oxidation in 1000 °C during 5 min after 200 h of nitriding.

**Figure 12:**
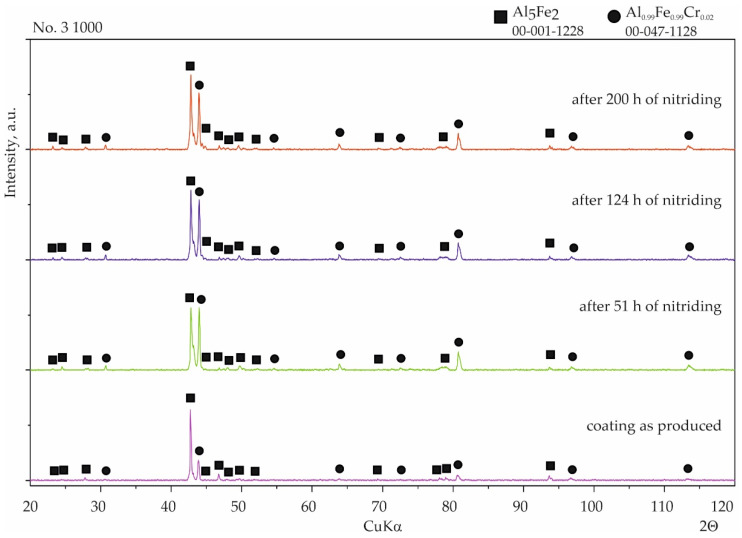
Qualitative X-ray structure analysis (XRD) of the Al-Si coating on 1.4541 steel produced at 1000 °C and 2 h from the bottom: in the initial state; after 51 h of nitriding; after 124 h of nitriding; and after 200 h of nitriding.

**Figure 13:**
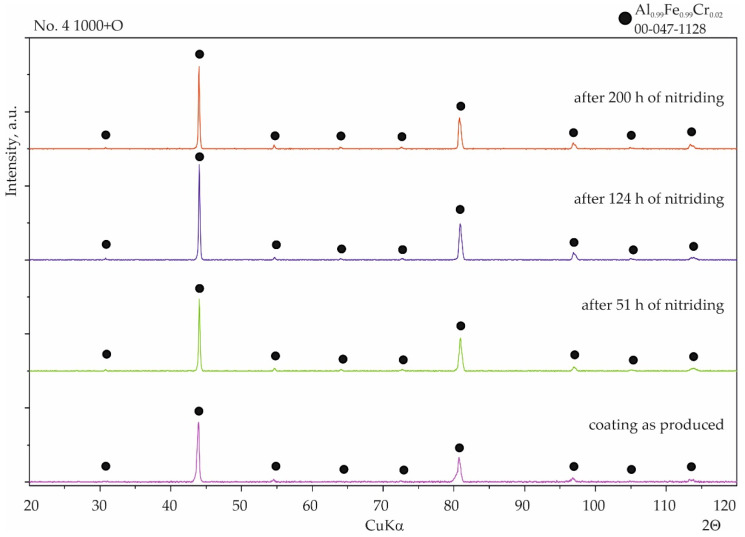
Qualitative X-ray structure analysis (XRD) of the Al–Si coating on 1.4541 steel produced at 1000 °C and 2 h with additional oxidation in 1000 °C during 5 min; from the bottom: in the initial state; after 51 h of nitriding; after 124 h of nitriding; and after 200 h of nitriding.

With coating No. 3, long-term exposure of the samples to the aggressive ammonia atmosphere had no effect on the surface morphology (Figure 11a). Identical results were obtained for the samples provided with coating No. 4, whereas with coating No. 2, many cavities were observed on the surface (Figure 11b). XRD phase analysis confirmed that no changes to the structure of these coatings had taken place (Figure 12 and Figure 13), indicating that the aluminide coatings were fully resistant to the aggressive nitriding atmosphere at elevated temperature.

## 4. Discussion

Generally, two types of aluminide coatings were produced (i.e., at temperatures of 800 and 1000 °C). Those produced at 800 °C (No. 1) should have a higher aluminum content on the surface as it is a process similar to that of the high activity pack cementation. The presence of the high aluminum Al_5_Fe_2_ phase confirmed this (Figure 8). Many previous studies have has shown that Al_5_Fe_2_ grows preferentially in the temperature range 700–800 °C, but that the AlFe phase dominates at a temperature of 1000 °C and above [30]. The predisposition to such a rapid growth probably results from the crystallographic structure of the Al_5_Fe_2_ phase, where there are a large number of aluminum vacancies along the c-axis of the orthorhombic cell structure of Al_5_Fe_2_, allowing for greater diffusion along this axis [31]. The process of coating formation at the higher temperature of 1000 °C (coating Nos. 3 and 4) was similar to low-activity, whereas a lower aluminum content was obtained on the surface. At the same time, a higher temperature promotes more rapid inward diffusion of aluminum and the formation of thicker coatings (Figure 4). The AlFe phase formed at the temperature of 1000 °C has the B2 structure.

Additional oxidation at 1000 °C of the coated samples (No. 2 and 4) resulted in the formation of a thin Al_2_O_3_ layer on the coating surface. This alumina layer is thermodynamically very stable and has a low growth rate, and many high-temperature alloys are designed to develop a “healing” Al_2_O_3_ layer, which remains protective up to 1350 °C [32]. Alumina is much more thermodynamically stable at high temperatures than chromium oxide Cr_2_O_3_ [33]. It is estimated that the limiting content of aluminum needed to form protective alumina scales at 800–1000 °C is approximately 14 at.% [34]. It should be emphasized that the oxidation in our experiment was carried out at 1000 °C, which, in the case of the coating No. 2 produced at the temperature of 800 °C, caused diffusion of the coating elements and structural changes (Figure 4a,c). After this annealing at 1000 °C, it was found that the AlFe phase was created. The aluminum content in the AlFe and Al_0.99_Fe_0.99_Cr_0.02_ phases was different: more aluminum was found in the Al_0.99_Fe_0.99_Cr_0.02_ phase. Aluminides based on AlFe with higher aluminum content exhibited improved oxidation and corrosion resistance compared to Al–Fe alloys with lower Al content, and were also lower in density by as much as 30–40% compared to steels and other commercial iron-based alloys [33]. The mechanical properties of the AlFe phase were also dependent on the aluminum content: although the yield strength increased with increasing aluminum content, the room-temperature tensile elongation of aluminides decreased with increasing aluminum content [20,35].

In the case of silicon and chromium, it has been observed that at a lower temperature of coating formation, there is a tendency for the Cr_3_Si phase to form precipitates on the surface (Figure 2b, Table 2) and in cross-section (Figure 4a). In contrast, a higher temperature of coating formation favors the dissolution of these elements in other Al–Fe phases (Table 2 and Table 3). The presence of chromium in the aluminide phases can be very beneficial. Chromium can improve the mechanical behavior without significantly affecting the corrosion properties of the material [36]. According to Klein and Baker [37], the addition of 5% Cr to a Fe_55_Al_45_ alloy significantly increases both the yield and fracture strengths. Additionally, aluminide diffusion coatings degrade by coating substrate interdiffusion, which results in a reduction in the subscale aluminum content and therefore decreases the coating lifetime. So far, the best alternatives for this type of coating seem to be diffusion aluminides with some chromium, which helps to retard aluminum loss by inward diffusion [38]. In the coatings presented in this article, chromium was dissolved in aluminides. The content of chromium was different depending on the phase in which it was dissolved. The highest chromium content was found in the AlFe phase (Figure 2c, Table 2—point 4 and Figure 4c, Table 3—point 5). This AlFe phase was identified in the coating produced at 800 °C and then oxidized at 1000 °C. In this case, it was the coating that was least resistant to corrosion in an ammonia atmosphere.

In summary, it can be stated that the coating structure on the samples with coatings No. 1, 3, and 4 did not change after 200 h of nitriding in the ammonia atmosphere. Iron nitrides were not observed, proving the heat resistance of these coatings. Only the samples with coatings produced at 800 °C with the additional oxidation at 1000 °C for 5 min (No. 2) deviated from the rest.

## 5. Conclusions

On the basis of the results obtained, the following conclusions can be formulated:

1. Austenitic stainless steel 1.4541 is not resistant to nitridation; this was confirmed by examinations after 51 h of nitriding.
2. The production of a diffusion coating on austenitic stainless steel 1.4541 is possible by the method of slurry cementation at temperatures of 800 and 1000 °C.
3. The technological parameters of the production process have a significant impact on the structure of silicide-aluminide coatings: the higher the coating formation temperature, the more homogeneous the structure and the greater its stability.
4. The four designed coating production processes resulted in the formation of four different phase compositions of the coatings. The coating produced at 800 °C was composed of Al_0.99_Fe_0.99_Cr_0.02_, Al_5_Fe_2_, and Cr_3_Si. The coating produced at 800 °C with additional oxidation at 1000 °C was composed of AlFe and a passive layer of Al_2_O_3_. The coating produced at 1000 °C was composed of Al_0.99_Fe_0.99_Cr_0.02_ and Al_5_Fe_2_. The coating produced at 1000 °C with additional oxidation at 1000 °C was composed of Al_0.99_Fe_0.99_Cr_0.02_ and a passive layer of Al_2_O_3_.
5. The diffusion coatings obtained enhanced the resistance of steel to high-temperature corrosion in a nitriding atmosphere. Besides the sample with the coating produced at 800 °C with additional oxidation at 1000 °C, silicon-aluminide coatings were resistant to aggressive ammonia during a 200 h treatment.
6. The phases Al_0.99_Fe_0.99_Cr_0.02_ and Al_5_Fe_2_ are resistant to high-temperature corrosion under a nitriding atmosphere, in contrast to the AlFe phase.

## Figures and Tables

**Table 1:** Technological parameters of the coating production.

Number of Coating Process	Annealing Temperature [°C]	Annealing Duration [h]	Oxidation: 5 min at 1000 °C (Heating Up to 1000 °C during 50 min)
No. 1	800	2	No
No. 2			Yes
No. 3	1000		No
No. 4			Yes

**Table 2:** The results of the EDS point analysis of the coating according to Figure 2.

Point of Analysis	Chemical Composition, at.%					Probable Phase
	Al	Si	Cr	Fe	Ni	
1	47.4	0.4	6.3	43.8	2.1	Al_0.99_Fe_0.99_Cr_0.02_
2	72.5	0.3	0.6	26.2	0.3	Al_5_Fe_2_
3	16.8	22.4	56.1	4.6	0.1	Cr_3_Si
4	45.6	1.0	10.6	40.0	2.7	AlFe
5	49.5	0.5	6.2	37.4	6.4	Al_0.99_Fe_0.99_Cr_0.02_
6	74.8	1.3	7.4	16.4	0.8	Al_5_Fe_2_
7	49.5	1.6	8.7	37.1	2.9	Al_0.99_Fe_0.99_Cr_0.02_

**Table 3:** The results of the EDS point analysis of the coating according to Figure 4.

Point of Analysis	Chemical Composition, at.%						Probable Phase
	O	Al	Si	Cr	Fe	Ni	
1		72.0	4.2	2.8	17.2	3.1	Al_5_Fe_2_
2		51.1	0.3	8.2	33.6	6.8	Al_0.99_Fe_0.99_Cr_0.02_
3		48.0	6.4	9.6	31.0	4.4	Al_0.99_Fe_0.99_Cr_0.02_
4		64.2	6.5	3.1	21.5	4.4	Al_5_Fe_2_
5		31.1	2.1	10.0	49.2	0.6	AlFe
6	52.0	47.2	0.0	0.3	0.5	0.0	Al_2_O_3_
7		43.3	5.5	9.0	37.4	4.9	Al_0.99_Fe_0.99_Cr_0.02_

**Table 4:** The results of the EDS/WDS point analysis of 1.4541 steel according to Figure 5.

Point of Analysis	Chemical Composition, at.%						
	C(WDS)	N (WDS)	O (WDS)	Si	Cr	Fe	Ni
1	1.0	8.9	7.6	1.9	18	57.7	4.9
2	1.1	10.9	17.3	0.6	14.6	51.9	3.6
3	1.0	13.8	3.6	0.2	1.7	75.0	4.7
4	1.1	7.0	13.9	1.5	16.5	53.9	6.1

## Data Availability

Data are contained within the article.

## References

[1] Venkataraman S., Jakobi D. (2017). Review on the Heat Resistant Stainless Steel Alloys Used for the Steam Methane Reformer Outlet Systems. Proceedings of the NACE International Corrosion Conference Proceedings.

[2] Kondrat’ev S.Y., Kraposhin V.S., Anastasiadi G.P., Talis A.L. (2015). Experimental observation and crystallographic description of M_7_C_3_ carbide transformation in Fe-Cr-Ni-C HP type alloy. Acta Mater..

[3] Kondrat’ev S.Y., Anastasiadi G.P., Ptashnik A.V., Petrov S.N. (2019). Kinetics of the High-Temperature Oxidation of Heat-Resistant Statically and Centrifugally Cast HP40NbTi Alloys. Oxid. Met..

[4] Picasso A.C., Lanz C.A., Lissarrague M.S., Garófoli A.D. (2016). Microstructure Evolution of a Nickel-Base Alloy Resistant to High Temperature during Aging. J. Miner. Mater. Charact. Eng..

[5] Tillack D.J., Guthrie J.E. (1998). Wrought and cast heat-resistant stainless steels and nickel alloys for the refining and petrochemical industries. Nickel Dev. Inst..

[6] Col A., Parry V., Pascal C. (2017). Oxidation of a Fe-18Cr-8Ni austenitic stainless steel at 850 °C in O_2_: Microstructure evolution during breakaway oxidation. Corros. Sci..

[7] Gheno T., Monceau D., Young D.J. (2013). Kinetics of breakaway oxidation of Fe-Cr and Fe-Cr-Ni alloys in dry and wet carbon dioxide. Corros. Sci..

[8] Agüero A., Baráibar I., Muelas R., Oskay C., Galetz M., Korner E. (2020). Analysis of an aluminide coating on austenitic steel 800HT exposed to metal dusting conditions: Lessons from an industrial hydrogen production plant. Int. J. Press. Vessel. Pip..

[9] Sequeira C. (2019). High Temperature Corrosion, Fundamentals and Engineering.

[10] Roberge P.R. (2019). Handbook of Corrosion Engineering.

[11] Kung S.C. (1990). High-temperature coating for titanium aluminides using the pack-cementation technique. Oxid. Met..

[12] Grabke H. (2007). Metal dusting. Corrosion by Carbon and Nitrogen.

[13] Sakidja R., Perepezko J.H., Calhoun P. (2014). Synthesis, Thermodynamic Stability and Diffusion Mechanism of Al_5_Fe_2_-Based Coatings. Oxid. Met..

[14] Wang J., Wu D.J., Zhu C.Y., Xiang Z.D. (2013). Low temperature pack aluminising kinetics of nickel electroplated on creep resistant ferritic steel. Surf. Coat. Technol..

[15] Duminica F.D., Vanden Eynde X., Mandy M., Nabi B., Georges C., Sturel T., Drillet P., Grigorieva R. (2020). Investigation of PVD thin films as hydrogen barriers in aluminized press hardened steels (PHS). Surf. Coat. Technol..

[16] Fryska S., Baranowska J., Przekop J., Suszko T. (2009). The properties of hard coating composed of S-phase obtained by PVD method. Adv. Manuf. Sci. Technol..

[17] Bhuvaneswaran N., Kamachi Mudali U., Shankar P. (2003). Characterization of aluminide coatings formed by diffusion alloying on nitrogen-containing type 316L stainless steels. Scr. Mater..

[18] Kochmańska A. (2012). Hot corrosion resistance properties of Al-Si coatings obtained by slurry method. Defect Diffus. Forum.

[19] Kochmańska A.E. (2018). Microstructure of Al-Si Slurry Coatings on Austenitic High-Temperature Creep Resisting Cast Steel. Adv. Mater. Sci. Eng..

[20] Deevi S.C. (2021). Advanced intermetallic iron aluminide coatings for high temperature applications. Prog. Mater. Sci..

[21] Tortorelli P.F., Natesan K. (1998). Critical factors affecting the high-temperature corrosion performance of iron aluminides. Mater. Sci. Eng. A.

[22] Mortazavi A.N., Esmaily M., Geers C., Birbilis N., Svensson J.E., Halvarsson M., Chandrasekaran D., Johansson L.G. (2020). Exploring failure modes of alumina scales on FeCrAl and FeNiCrAl alloys in a nitriding environment. Acta Mater..

[23] Meka S.R., Bischoff E., Schacherl R.E., Mittemeijer E.J. (2012). Unusual nucleation and growth of γ iron nitride upon nitriding Fe-4.75 at. % Al alloy. Philos. Mag..

[24] Le N.M., Schimpf C., Biermann H., Dalke A. (2021). Effect of Nitriding Potential KN on the Formation and Growth of a “White Layer” on Iron Aluminide Alloy. Metall. Mater. Trans. B Process Metall. Mater. Process. Sci..

[25] Zhang Z., Li X., Dong H. (2015). Plasma-nitriding and characterization of FeAl40 iron aluminide. Acta Mater..

[26] Berrached I., Rabahi L., Gallouze M., Kellou A. (2019). Nitriding effect on structural stability and magnetic properties of FeAl alloys: DFT study. J. Magn. Magn. Mater..

[27] Tamarin Y. (2002). Protective Coatings for Turbine Blades.

[28] Kepa T., Pedraza F., Rouillard F. (2020). Intermetallic formation of Al-Fe and Al-Ni phases by ultrafast slurry aluminization (flash aluminizing). Surf. Coat. Technol..

[29] Kochmański P., Baranowska J., Fryska S. (2019). Microstructure of low-temperature gas-carbonitrided layers on austenitic stainless steel. Metals.

[30] Kobayashi S., Yakou T. (2002). Control of intermetallic compound layers at interface between steel and aluminum by diffusion-treatment. Mater. Sci. Eng. A.

[31] Shahverdi H.R., Ghomashchi M.R., Shabestari S., Hejazi J. (2002). Microstructural analysis of interfacial reaction between molten aluminium and solid iron. J. Mater. Process. Technol..

[32] Zhang Z.G., Gesmundo F., Hou P.Y., Niu Y. (2006). Criteria for the formation of protective Al_2_O_3_ scales on Fe-Al and Fe-Cr-Al alloys. Corros. Sci..

[33] Deevi S.C., Sikka V.K. (1996). Nickel and iron aluminides: An overview on properties, processing, and applications. Intermetallics.

[34] Pint B.A., Leibowitz J., Devan J.H. (1999). Effect of an oxide dispersion on the critical Al content in Fe-Al alloys. Oxid. Met..

[35] Liu C.T., Stringer J., Mundy J.N., Horton L.L., Angelini P. (1997). Ordered intermetallic alloys: An assessment. Intermetallics.

[36] Ortega Y., de Diego N., Plazaola F., Jiménez J.A., del Río J. (2007). Influence of Cr addition on the defect structure of Fe-Al alloys. Intermetallics.

[37] Klein O., Baker I. (1992). Effect of chromium on the environmental sensitivity of FeA1 at room temperature. Scr. Metall. Mater..

[38] Agüero A., Gutiérrez M., Korcakova L., Nguyen T.T.M., Hinnemann B., Saadi S. (2011). Metal dusting protective coatings. A literature review. Oxid. Met..

